# LAMP5 Fine-Tunes GABAergic Synaptic Transmission in Defined Circuits of the Mouse Brain

**DOI:** 10.1371/journal.pone.0157052

**Published:** 2016-06-07

**Authors:** Marie-Catherine Tiveron, Corinne Beurrier, Claire Céni, Naly Andriambao, Alexis Combes, Muriel Koehl, Nicolas Maurice, Evelina Gatti, Dhoher Nora Abrous, Lydia Kerkerian-Le Goff, Philippe Pierre, Harold Cremer

**Affiliations:** 1 Aix-Marseille University, Centre National pour la Recherche Scientifique, IBDM, Developmental Biology Institute of Marseille, UMR 7288, 13009, Marseille, France; 2 Centre d’Immunologie de Marseille-Luminy, Aix-Marseille Université UM2, Inserm U1104, CNRS UMR7280, 13288, Marseille, France; 3 Neurogenesis and Physiopathology Group, INSERM U862, NeuroCentre Magendie, 33076, Bordeaux, France; University of Nebraska Medical Center, UNITED STATES

## Abstract

LAMP5 is member of the LAMP family of membrane proteins. In contrast to the canonical members of this protein family, LAMP1 and LAMP2, which show widespread expression in many tissues, LAMP 5 is brain specific in mice. In *C*. *elegans*, the LAMP5 ortholog UNC-46 has been suggested to act a trafficking chaperone, essential for the correct targeting of the nematode vesicular GABA-transporter UNC-47. We show here that in the mouse brain LAMP5 is expressed in subpopulations of GABAergic forebrain neurons in the striato-nigral system and the olfactory bulb. The protein was present at synaptic terminals, overlapping with the mammalian vesicular GABA-transporter VGAT. In *LAMP5*-deficient mice localization of the transporter was unaffected arguing against a conserved role in VGAT trafficking. Electrophysiological analyses in mutants showed alterations in short term synaptic plasticity suggesting that LAMP5 is involved in controlling the dynamics of evoked GABAergic transmission. At the behavioral level, *LAMP5* mutant mice showed decreased anxiety and deficits in olfactory discrimination. Altogether, this work implicates LAMP5 function in GABAergic neurotransmission in defined neuronal subpopulations.

## Introduction

Trafficking of cellular components in a coordinated manner represents a major challenge for all cells. This problem is particularly evident in the case of neurons where proteins, lipids and RNA have to be transported over large distances along axons or into highly complex dendritic arborizations to reach pre- or postsynaptic sites [1]. Once at the synapse, the situation complexifies further, as neuron-to-neuron communication critically depends on efficient vesicular loading and sorting, high-speed exocytosis and rapid recycling of cellular material and neurotransmitter [2]. Finally, synaptic communication is not a linear transformation of an electric stimulus into neurotransmitter release, but a locally modifiable process, whose plasticity underlies learning and memory [3].

Work over the past decades demonstrated that many mechanisms managing the cellular traffic of molecules are shared between neurons and other cells [4,5]. For example, the clathrin system is widely used in plants, yeast and animals to control endocytosis and intracellular trafficking of defined targets in concert with adaptor proteins that specify the cargo [6]. In neurons, clathrin-mediated endocytosis represents the predominant mode of synaptic vesicle protein internalization [7–9]. However, due to their particular properties neurons need additional and unique trafficking systems that address their specific needs.

Lysosome Associated Membrane Proteins (LAMPs) represent a family of membrane proteins sharing sequence and structural homology. Prototypic members of this family like LAMP1 and LAMP2 are widely expressed in many tissues and have been implicated in a variety of intracellular trafficking events, often, although not exclusively, associated to lysosomal transport [10–12].

Based on sequence and structural homology LAMP5 (BAD-LAMP/C20orf103) has been classified as a LAMP-family member. However, in contrast to canonical members of this family, that show widespread expression [11], LAMP5 is, at least in rodents, strictly confined to the brain where it appears at postnatal stages coincidently with synaptogenesis [11,12]. In neurons, LAMP5 accumulates in distinct intracellular vesicles that do not contain any known markers of classical intracellular transport pathways and are not targeted to the endosomal/lysosomal compartment [13]. Interestingly, the LAMP5 orthologue in *C*. *elegans*, *unc-46*, has been shown to be specifically expressed in synaptic vesicles of GABAergic neurons. Mutations in *unc-46* interfere with GABA neurotransmission [14]. Based on the observations that in *unc-46* mutants the Vesicular GABA Transporter (VGAT) is miss-localized to non-vesicular compartments and that loading of synaptic vesicles with GABA is deficient, it was proposed that UNC-46 functions as a trafficking chaperone that targets VGAT to synaptic vesicles [14].

Here we show that in mice LAMP5 protein is specifically expressed in subpopulations of GABAergic neurons where it localizes at synaptic terminals, overlapping with VGAT. However, in the absence of LAMP5, VGAT distribution was not affected, arguing against a constitutive role of the protein as a trafficking chaperone for this transporter. In contrast, alterations in pre-synaptic plasticity and subtle behavioral alterations in mutant mice point towards with a fine-tuning function of LAMP5 in evoked GABAergic synaptic transmission.

## Materials and Methods

### Animals

Animal experiments were carried out in accordance to European Communities Council Directive and approved by French ethical committees (Comité d’Ethique pour l’expérimentation animale n°14; permission number: 62–12112012). For all experiments, animals were group-housed (3–6 per cages) under a 12 hours light-dark cycle in a controlled environment. Food and water were provided ad libitum.

### Generation of LAMP5 mutant mice

*LAMP5* conditional mutant mice were generated by the KO-KI Booster platform, CIPHE, Marseille (http://www.celphedia.eu/en/centers/ciphe). The detailed procedure can be given on request. Briefly, the BAC RP-330F10 clone isolated from C57BL/6 genome containing *LAMP5* locus was used to construct the targeting vector. LoxP sites were introduced upstream of exon2 and downstream of exon 5 therefore flanking the exons carrying the majority of the coding sequence. This construct was electroporated into C57Bl/6 embryonic stem cells and clones were analyzed by Southern blots using 5’ and 3’ probes. Two clones that underwent homologous recombination were used to produce chimeric mice. Recombinant heterozygous mice carrying the floxed allele *LAMP5*^*flox*^ were either crossed together to produce homozygotes *LAMP5*^*flox/flox*^ mice or crossed with a Cre deleter mice (Swenk et al. 1995) that express Cre during early embryogenesis giving rise to heterozygous mice *LAMP5*^*+/-*^ carrying a deleted *LAMP5* allele, *LAMP5*^*-*^. Mutant mice were obtained after crossing heterozygotes, their wild type littermates were used as control.

### In situ hybridization and immunohistochemistry

Animals were deeply anesthetized with an overdose of xylasin/ketamine, then intracardially perfused with 4% paraformaldehyde (wt/vol) in PBS. The brain were dissected out and further fixed overnight at 4°C in the same fixative. In situ hybridization was performed on cryostat 15 μm sections as described previously [15] using the IMAGE clone 2588577 to make an antisense RNA probe [13]. For immunohistochemistry, 50 μm floating sections were processed as described previously [16] using primary antibodies against LAMP5 (mAb 34.2, rat IgG, 1:400, [17], GAD65 (Goat IgG, 1:1000, R&D Systems), VGlut1 (rabbit IgG, 1:500, Synaptic Systems), VGAT (rabbit IgG, 1:200, kind gift of B. Gasnier, [18] followed by incubation with corresponding secondary antibodies, Alexa 555-conjugated antibody to rat and Alexa 488- conjugated antibodies to rabbit or goat (1:500, InVitrogen). Confocal images were taken using confocal microscope LSM 510 (Zeiss). Co-localization quantification of LAMP5 with GAD65 or VGAT was obtained from automated image analysis using JacoP plugin on ImageJ (Bolte and Cordelières, 2006). Quantification was done on 4 pictures taken on different sections from 2 experiments on one wild type animal. For electron microscopy immunolabeling, animals were perfused with 3% PFA; 0.2% glutaraldehyde, 50 μm vibratome sections were incubated with rat mAb34.2 then with anti-rat IgG conjugated to ultrasmall nanogold particles (Aurion). After a silver enhancement step (Aurion R-Gent SE-EM), these pre-embedded immunolabeled sections were then processed for transmission electron microcopy. Grids were examined using an electron microscope Zeiss EM 912 and digital images acquired with CCD camera Gatan Bioscan model 792.

### Tissues, Quantitative PCR and immunobloting

Mice were killed by decapitation and brains were dissected out. OB were recovered first then the other tissues -striatum, pallidum, Substantia nigra pars reticulata (SNr)- were micro dissected from 300 μm thick vibratome brain sections. All tissues were snap frozen and stored at -80°C until processed for RNA extraction or used for immunobloting. For quantitative PCR, RNA was extracted using the miRNAeasy kit (Qiagen) and cDNA was prepared using superscript III reverse transcriptase (Invitrogen). Quantitative PCR was performed on a BioRad CFX system using SYBR GreenER qPCR SuperMix (Invitrogen). *Hprt* was used as a reference gene. The oligonucleotide used for amplification were LAMP5 forward (ACTGTCACCATGATCCTGTCC), LAMP5 reverse (CTGGGGATCTGCACTTGATT), hprt forward (CTGGTGAAAAGGACCTCTCG) and hprt reverse (TGGCAACATCAACAGGACTC). The experiment was done twice. Quantification and error bars are generated automatically by BioRad software from 3 wells per gene per sample.

For immunoblot analysis, tissues were lysed in 50mM Tris pH7.5, 150mM NaCl, 1mM EDTA, 1% NP40, 0.25% NaDOC in presence of protease inhibitors (Complete, Roche). 20 μg of protein were immunobloted after separation on 4–12% Bis-Tris acrylamide gels (Novex; Life Technologies). First, LAMP5 protein was detected with rat mAb34.2 (1:400) as primary antibody, anti-rat Fab conjugated to horseradish peroxidase (HRP) as secondary antibody and visualized by Enhanced ChemiLuminescent detection kit (GE Healthcare). For normalization, the blot was stripped and Actin protein was detected using a mouse IgG2a anti-Actin antibody (1:2000; SIGMA) and an anti-mouse IgG2A conjugated to HRP. Quantification of blots was performed by measuring signal intensity using ImageJ.

### Forebrain electroporation and analyses

Plasmid DNA was injected in the ventricule of P1 pups and electroporated in the lateral wall to target olfactory bulb interneurons precursors as described in Boutin et al, 2008. pCAGGS GFP plasmid was injected either alone in CD1 wild type P1 pups or together with pCAGGS-Cre plasmid (kind gift X. Morin) in *LAMP5*^*flox/+*^ or *LAMP5*^*flox/flox*^ pups. Twenty eight days post electroporation the bulb were processed for immunohistochemistry as described above or directly analyzed to assess spine density. The quantification of the latter was performed on 26 (control) and 27 (mutant) dendrites from 4 animals for each condition.

### OB subcellular fractionation and immunoisolation of synaptic vesicles

12 adult mice were killed by decapitation and OB were dissected on ice and placed into an ice cold sucrose/HEPES buffer (0.32M sucrose; 4mM HEPES/KOH buffer, pH 7.3). Synaptic vesicles were purified as described (Huttner et al. J. Cell Biol, 1983) after subcellular fractionation. Briefly, the OB were homogenized in ice cold sucrose/HEPES buffer (HSB) in a glass-Teflon homogenizer. The pellet representing the nuclear fraction was discarded after centrifugation at 1000 g for 10 min and the supernatant (S1) was further centrifuged at 12500 g for 15 min. The pellet (P2) was resuspended in 10 ml HSB and centrifuged again at 12500 g for 15 min. The resulting pellet (P2’) constituting the synaptosome fraction was resuspended 0.5ml of HBS. This fraction was then lysed osmotically in 5 ml of ice cold H_2_O, homogenized and centrifuged at 25000 g for 20 min. The supernatant (LS1) representing the synaptic vesicles fraction was used for immunoisolation of the vesicles. Protein G Plus-agarose beads (Santa Cruz) were coupled to mouse IgG against VGAT (Synaptic Systems) or against NeuN (Chemicon) for control. The conjugated beads were incubated with LS1 fraction corresponding to 200 μg of proteins, overnight at 4°C in presence of protease inhibitors (Complete, Roche). After several washes in PBS, the bound synaptic vesicles were eluted in Laemli buffer. These samples were immunobloted after separation on 4–12% Bis-Tris acrylamide gels (Novex; Life Technologies). The presence of LAMP5 on the immunoisolated vesicles fraction was then detected using rat mAb34.2 and the blot was processed as described before.

### Electrophysiology

Electrophysiological recordings from 4 to 6 week-old LAMP5 mutant and wild-type (WT) littermate mice were interleaved whenever possible. Parasagittal slices (250 μm) containing the striatum and the GP were cut with a VT1000 S vibratome (Leica, Wetzlar, Germany) in ice-cold high-choline artificial cerebro-spinal fluid (ACSF) as previously described [19,20] and maintained at room temperature until recordings. Slices were then transferred to a submersion recording chamber and continuously perfused (3 ml/min) with standard ACSF containing (in mM): 126 NaCl, 2.5 KCl, 1.2 NaH_2_PO_4_, 1.2 MgCl_2_, 2.4 CaCl_2_, 25 NaHCO_3_ and 11 glucose, gassed with 95% O_2_ / 5% CO_2_ and warmed to 33°C (pH 7.4). All recordings were performed in ACSF + 20 μM CNQX (Sigma-Aldrich), to block glutamatergic transmission, in voltage-clamp at a holding potential of -50 mV with electrodes (4–6 MΩ) containing (in mM): 140 CsCl, 10 NaCl, 0.1 CaCl, 10 HEPES, 1 EGTA, 2 Mg-ATP et 0.5 Na-GTP, pH = 7.25, 270–290 mOsm/L. Miniature inhibitory post-synaptic currents (mIPSCs) were recorded in the presence of 1 μM tetrodotoxin (TTX), a blocker of voltage-dependent sodium channels. IPSCs were evoked every 10 seconds in the GP by a stimulating electrode (parallel bipolar electrode, # PBSC0275, FHC) placed in the striatum. For paired-pulse ratios (PPRs), two stimuli were delivered from 20 to 150 ms interstimulus interval (ISI) and PPRs were calculated as IPSCs2 amplitude / IPSCs1 amplitude (10 sweeps average per ISI). Synaptic repetitive stimulation was induced by trains of stimulation (10 pulses at 20 Hz). Neurons were visualized on an upright microscope (Nikon Eclipse FN1) equipped with DIC optic using a x40 water-immersion objective. Data were collected with a MultiClamp 700B amplifier (Molecular Devices).

Data are presented as mean ± standard error of the mean (SEM). Statistical significance was assessed using a nonparametric test (test of Mann-Whitney, MW) (SigmaStat, v3.1) and a Kolmogorov-Sminov test for the cumulative probability distributions. A significance of *p* < 0.05 was required for rejection of the null hypothesis.

### Behavioral studies

All behavioral studies were carried out with wild type and homozygous male littermates derived from crossing heterozygous *LAMP5*^*+/-*^ mice. Mice cohorts grouping individuals born within two weeks and between 12 to 16 weeks of age were used for rotarod, catalepsy and odor discrimination tests. For locomotor activity and Elevated Plus maze tests, two independent cohorts were used, one between 12–14 weeks of age and another between 18–20 weeks. As inter-cohort statistical analysis did not show any difference, test results from all individuals were pooled. All studies were performed blind to genotype.

#### Locomotor activity

Locomotion was assessed in photocell-based activity chambers. Each mouse was placed in a Plexiglas activity box (18.2x12x22 cm) equipped with infrared sensors, allowing measurement of ambulatory locomotor activity. Each box was connected to a computer by an electronic interface (Imetronic). Spontaneous activity was measured for 2 hours.

#### Rotarod test

This test assesses motor co-ordination and balance. In this test, mice have to keep their balance on a rotating rod under continuous acceleration from 4 rpm to 40 rpm in 300 seconds. Latency of each mouse to fall from the rod is recorded. The test was carried out following the protocol described in “European Mouse Phenotyping resource of Standardised Screen” (EMPReSS; "http://empress.har.mrc.ac.uk/). Briefly, after being trained to stay on the rotating rod set at a stable speed (4 rpm) for one minute, mice started their test session. This session consisted of 3 trials separated by 10 minutes inter trial intervals. The mean of latency over the three trials gives the latency reported per animal.

#### Elevated Plus Maze test (EPM)

Anxiety related behavior was measured using the EPM test as described previously [21]. This test uses the mice natural fear of height and open space and its preference to dark/enclosed space. Briefly, each mouse was placed in the center of an elevated maze with four arms (two open and two enclosed) that are arranged to form a plus shape, and was allowed to freely explore the maze for 5 min. The mouse path was tracked and automatically analyzed (Videotrack, Viewpoint).

#### Haloperidol-induced catalepsy

Catalepsy was measured using the catalepsy bar test [22,23]. Briefly, after intraperitoneal Injection of 1 mg per kg of haloperidol (Haldol 5mg/*ml*, *Janssen-Cilag*), mice were placed in an individual Plexiglass cage. Cataleptic state was measured by placing the front paws of the animal on a horizontal steel bar (4cm high, 15cm wide). The length of time the animals stayed in this position was recorded to a maximum of 300 seconds. After each test, animals were placed back to their Plexiglass cage. Measurements were conducted 15 min after haloperidol administration and then, animals were tested every 20 min for 155 min.

#### Odor discrimination test

The protocol used is described in [24]. Odor discrimination is based on a habituation/dishabituation task that consists of four consecutive presentations of a habituation odorant (Oh) followed by one presentation of a test odorant (Ot). The time that the mouse spent sniffing the odors at each presentation was recorded. Significant increase of the investigation time of the Ot compared to the fourth Oh presentation indicates discrimination. Three sets of odors were used isoamylbutyrate/heptanol, hexanol/limonene^-^ and isoamylacetate/carvone. Each odorant was diluted 1/1000 in mineral oil.

### Quantification and Statistical analysis

Results are expressed as mean ± SEM. In the Rotarod and EPM assays, differences between groups were assessed by using Kruskal-Wallis statistical test. In the olfaction test, within-subjects analyses of variance (ANOVA) were performed across the four habituation trials, with trial number as the within-subjects factor and genotype as a between-subjects factor to assess habituation. Subsequently, Wilcoxon pairwise comparisons were performed to determine whether mice of each genotype habituated to the habituation odor (Oh) as indicated by a significant decrease in investigation time between the first (Oh1) and the fourth (Oh4) odor presentation. To assess odor discrimination, Wilcoxon pairwise comparisons were performed to determine whether the investigation time elicited by Ot was significantly increased from that elicited by Oh during the fourth odor presentation. All data analysis was performed with R Software (The R Project for Statistical Computing).

## Results

### LAMP5 is synaptically localized in subpopulations of GABAergic neurons

**Fig 1:**
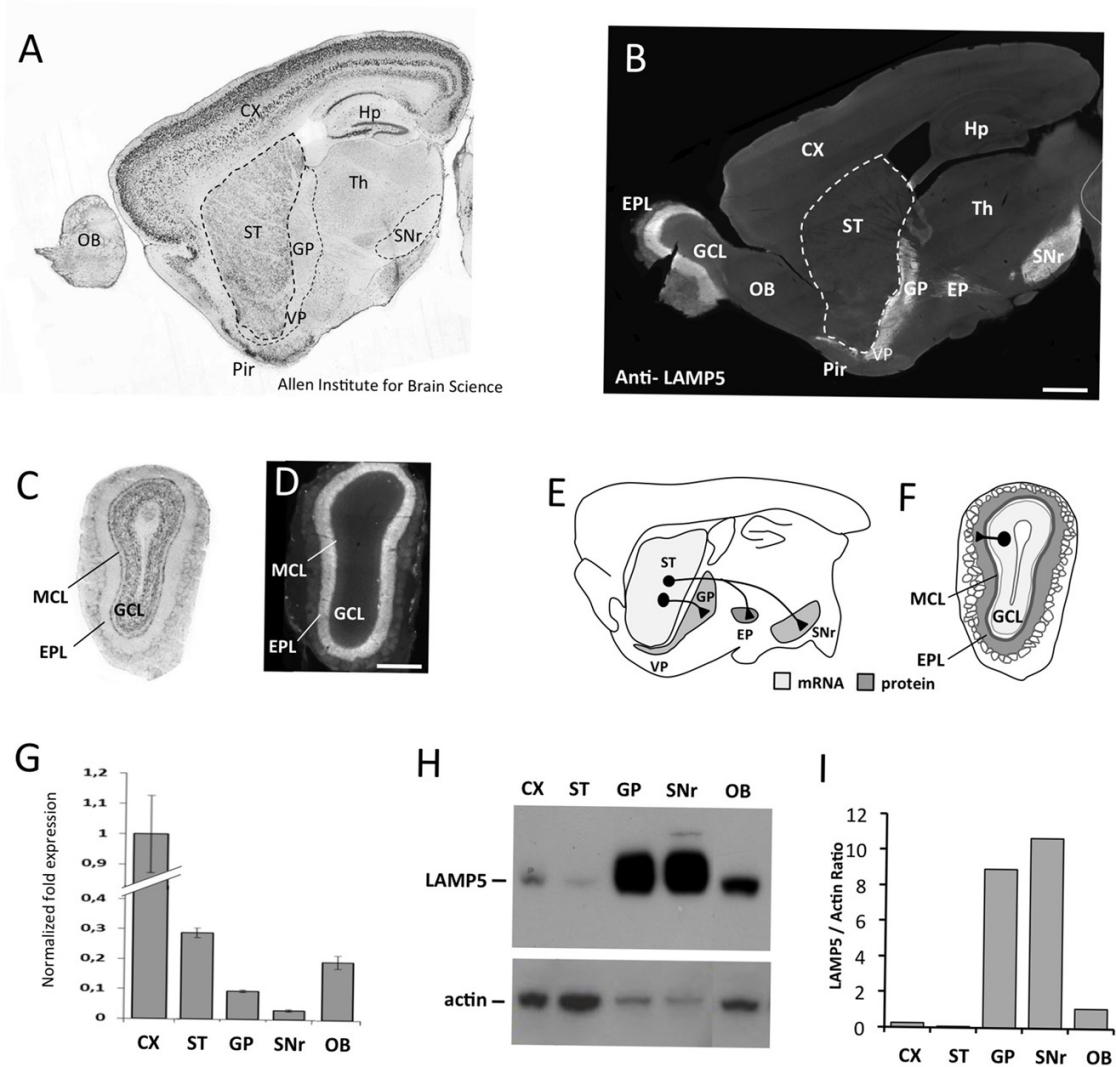
Differential expression of *LAMP5* mRNA and protein in the brain. (A,C) In situ hybridization and (B,D) immunohistochemistry for LAMP5 on sagittal brain sections (A,B) and on coronal olfactory bulb (OB) sections (C,D). Strongest expression of *LAMP5* mRNA is found in the neocortex (CX), piriform cortex (Pir), hippocampus (Hp), striatum (ST) and the granular cell layer (GCL) of the OB. LAMP5 protein is strongly present in the Globus Pallidus/Ventral Pallidum complex (GP/VP), the Substantia Nigra pars reticulata (SNr) and the entopeduncular nucleus (EP), that are the main output structures of the striatal GABAergic projection neurons, and in the external plexiform layer (EPL), in which granule cells positioned in the granule cell layer (GCL) form GABAergic synapses. (E-F) Schematic representation of *LAMP5* mRNA (light grey) and protein (dark grey) expression in the rodent forebrain (E) and OB (F). (G) qRT-PCR analysis of *LAMP5* expression in different brain tissue samples. Coherent with immunohistochemical stainings, strongest expression of *LAMP5* mRNA is detected in the cortex, striatum and the OB. (H) Western blotting and its quantification (I) demonstrates that LAMP5 protein is strongly expressed in GP and SNr while striatal tissue (ST) and cortex show only weak signal. Th: thalamus; Scale bar: 1 mm in A,B; 0.5 mm in C,D.

In situ hybridization (from Allen Brain Atlas, URL: http://mouse.brain-map.org/) showed strongest expression of *LAMP5* mRNA in the forebrain in the neocortex, piriform cortex, hippocampus and striatum (Fig 1A). In addition, the mitral and granule cell layers of the olfactory bulb (OB) showed high signal levels (Fig 1C). Interestingly, the LAMP5 specific monoclonal antibody mAb 34.2 [17] showed a largely non-overlapping pattern. LAMP5 immunoreactivity was absent from the cortex, but confined to the globus pallidus (GP)/ventral pallidum (VP) complex as well as the substantia nigra pars reticulata (SNr, Fig 1B). In the OB the external plexiform layer (EPL) showed strong LAMP5 immunoreactivity (Fig 1D). This specific distribution was confirmed using microdissected tissue. qRT-PCR analyses using primers within the coding region of *LAMP5*, and including the peptide sequence used for immunization, detected high levels of *LAMP5* mRNA in the cortex and the striatum, while expression was low in GP and SNr. (Fig 1G). In agreement with the histological data, western blotting of tissue extracts showed very low levels of LAMP5 immunoreactivity in the cortex and striatum but strong expression in GP and SNr (Fig 1H and 1I). OB was taken as a whole and showed expression of both, mRNA and protein (Fig 1G, 1H and 1I).

**Fig 2:**
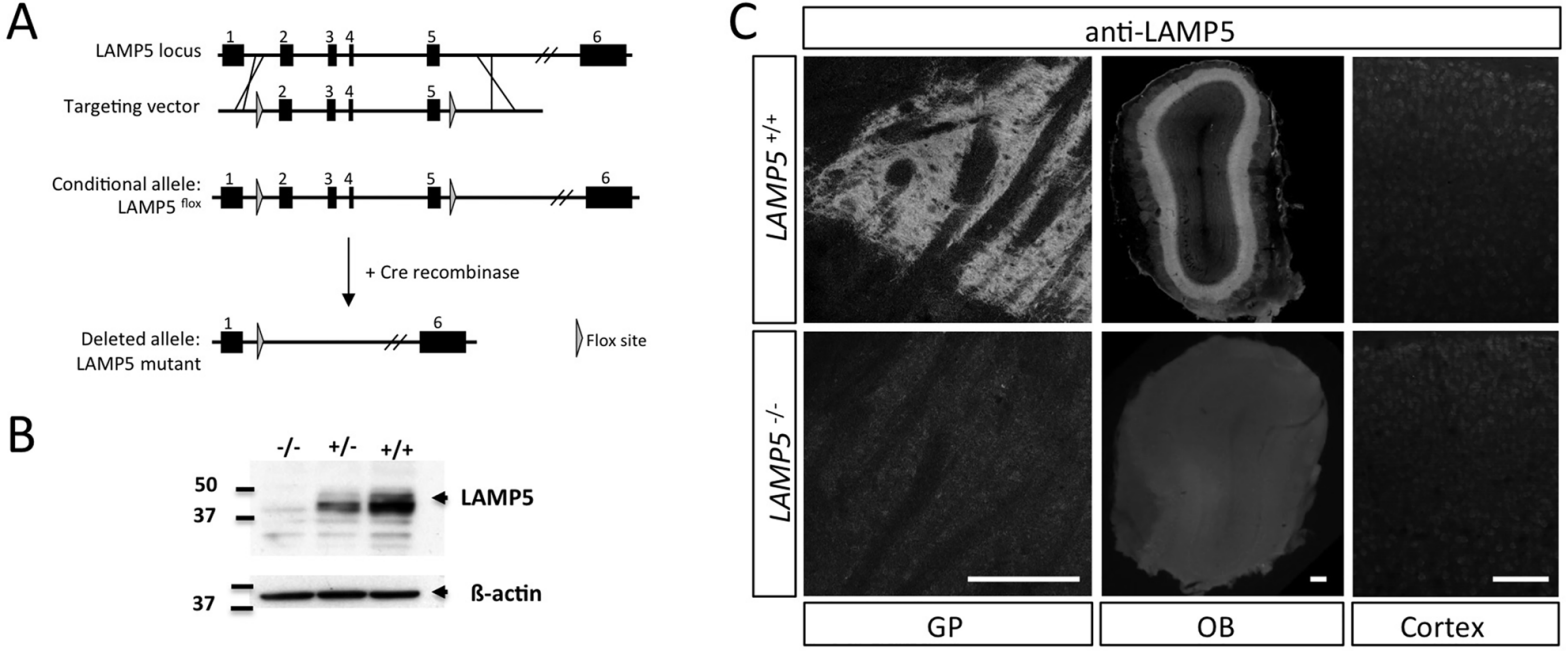
*LAMP5* deficient mice validate mAb 34.2 anti-LAMP5 antibody specificity. (A) Targeting strategy to generate a *LAMP5* deficient mouse line. The targeting vector was designed to remove exons 3–5 after CRE-induced recombination, leading to a null allele. *LAMP5* null animals are viable and therefore used for most analyses. (B) Western blot of KO (-/-), heterozygote (+/-) and wild type (+/+) mouse brains demonstrates absence of protein in homozygous KOs. (C) Immunohistochemistry with mAb 34.2 anti-LAMP5 antibody on *LAMP5*^*+/+*^ and *LAMP5*^*-/-*^ tissue sections of GP and OB validates the absence of LAMP5 protein in KOs. Cortex was always negative. Scale bars: C, 200 μm for GP and OB; 100 μm for cortex.

The pattern of LAMP5 immunoreactivity based on the m34.2 antibody was in partial contradiction to previous results obtained with a polyclonal rabbit serum [13]. We generated *LAMP5*-deficient mice by flanking exons 2–5 with loxP sites and breeding this allele to mice expressing the CRE recombinase ubiquitously at the early embryonic stage (Fig 2A, [25]. Western blot analyses of brain extracts demonstrated the total absence of mAb 34.2 immunoreactivity in homozygous KOs and the expected dose reduction in heterozygotes (Fig 2B). Specificity was further confirmed by immunofluorescence analyses of brain sections, showing that two structures with highest signal levels, GP and OB (see Fig 1) were negative in KOs. Cortex showed no specific signal in the WT (Fig 2C). We conclude that m34.2 is LAMP5 specific.

Altogether, the above results indicate that *LAMP5* mRNA was present in subpopulations of both GABAergic and glutamatergic neurons in the forebrain. However, in the cortex, hippocampus and the OB mitral cell layer, LAMP5 protein was absent. The situation was different for the striatal *LAMP5* mRNA positive cell populations. Here, LAMP5 protein was non-detectable in the striatum itself, but was strongly expressed in the main output structures for GABAergic striatal neurons, the GP/VP and SNr (Fig 1E). Since these structures were negative for *LAMP5* mRNA, this indicated synaptic localization of LAMP5 protein in striatal neurons. In the OB, the protein was confined to the EPL, the layer in which GABAergic interneurons of the granule cell layer form synapses with mitral and tufted cells (Fig 1F; [26]. This, again, suggested synaptic targeting of LAMP5 protein.

**Fig 3:**
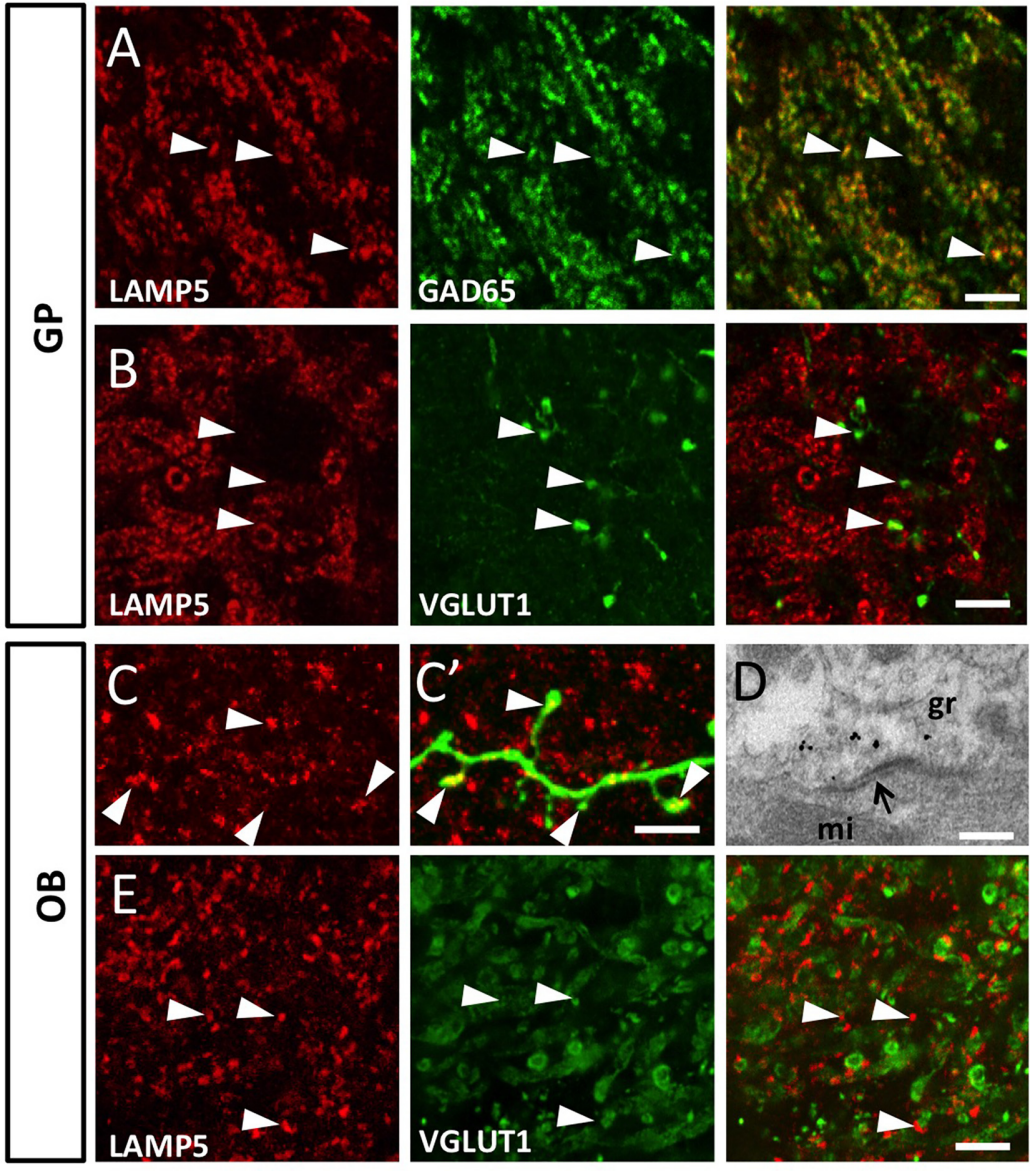
LAMP5 is specifically expressed in GABAergic synapses. (A) Immunofluorescence labeling for LAMP5 and GAD65 proteins demonstrates co-labelling in GABAergic axon terminals in the GP (arrowheads). (B) LAMP5 immunoreactivity never overlaps with vGLUT1 (arrowheads), thus it is absent from glutamatergic synapses. (C, C') In the OB, LAMP5 is localized on the synapses (arrowheads) of newly generated granule neurons, labeled with GFP by in vivo brain electroporation. (D) Electron microscopy immunogold labeling validates synaptic localization of LAMP5. Electron dense gold particles are always associated with symmetric synaptic densities (arrow) typical of GABAergic synapses formed by granule neurons onto mitral cell dendrites. (E) Like in the GP, glutamatergic synapses in the OB are devoid of LAMP5 staining (arrowheads). Scale bars: 5 μm in A,B,C,E, F; 0,1 μm in D.

We aimed at validating this distribution at higher resolution using immunofluorescence and electron microscopy. In both, the GP and the EPL of the OB, LAMP5 immunostaining showed the typical punctate aspect indicative of synaptic localization (Fig 3A–3C and 3E). In the GP co-staining for glutamate-decarboxylase 65 (GAD65), a GABA-synapse specific protein, revealed that 69.1 +/- 3.9% of the LAMP5 positive punctae labeled also for this presynaptic marker (n = 4; Fig 2A). Staining for the glutamatergic synapse marker VGLUT1 was always non-overlapping with LAMP5 (Fig 3B and 3E).

Next we combined immunofluorescence for LAMP5 with in vivo brain electroporation in the OB neurogenic system, which allows visualization of synaptic contacts of OB granule cells at high resolution. A GFP expression construct was electroporated in neural stem cells of the lateral ventricular wall at postnatal day 1 (P1) as described before [27,28]. Twenty one days later GFP positive GABAergic granule neurons derived from the labeled stem cells were found in the OB and formed dendritic spines contacting shafts of mitral cells within the EPL [26]. The majority of these newly formed spines were immunoreactive for LAMP5 (Fig 3C and 3C’). To further increase resolution, we used immunogold electron microscopy. Gold particles linked to the LAMP5 antibody were always associated with the typical synapses that granule neurons in the OB form on the apical dendrites of mitral cells (Fig 3D). Moreover, like in the GP, labeling of LAMP5 and the glutamatergic marker VGLUT1 showed little to no co-localization in the OB (Fig 3E). Altogether, these data indicate that sub-populations of forebrain GABAergic neurons in the striatum and OB express LAMP5 specifically at their synaptic contacts.

### LAMP5 is not essential for correct localization of VGAT

**Fig 4:**
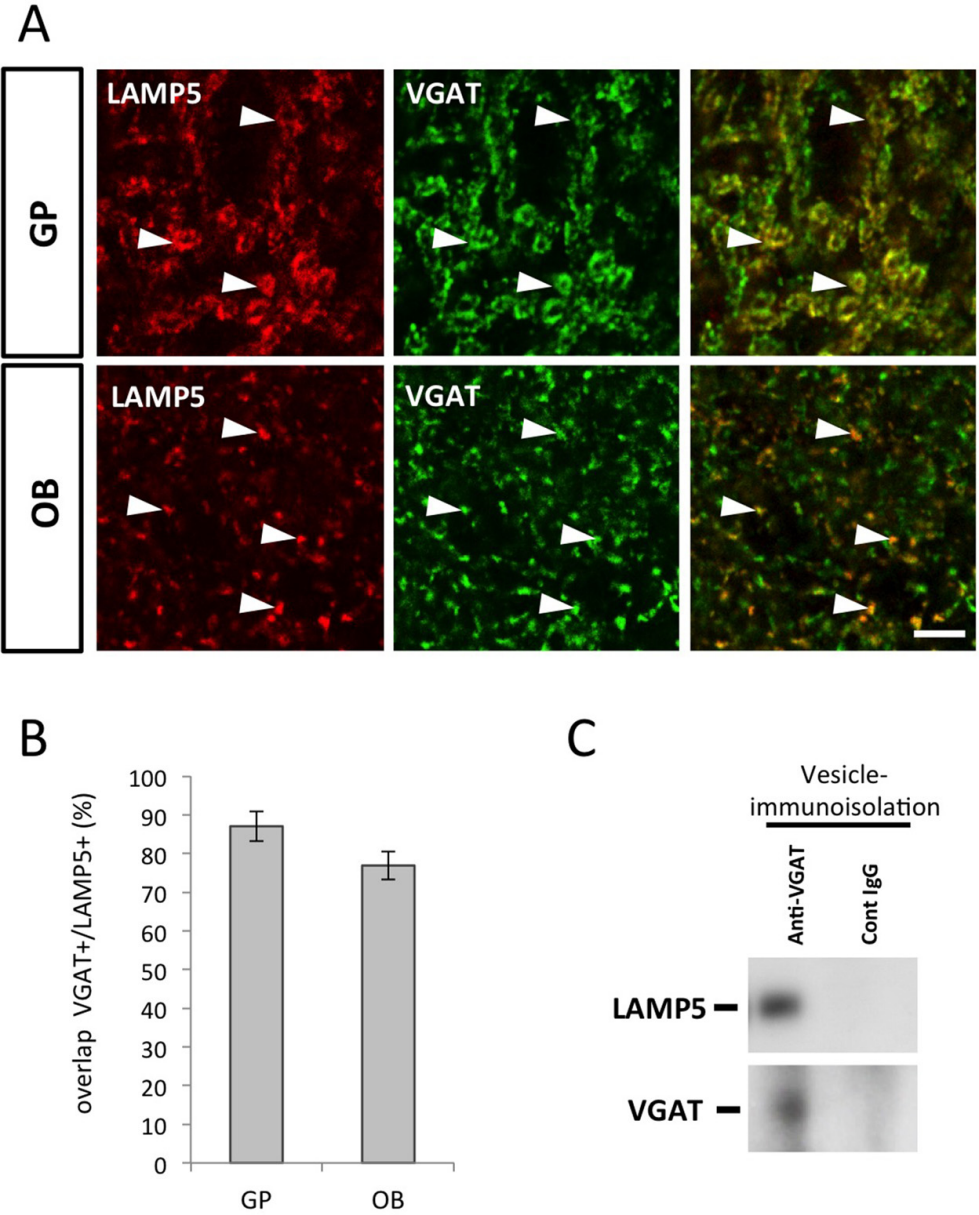
LAMP5 is present on VGAT positive synaptic vesicles. (A) Histological sections of the GP and the OB immunostained for LAMP5 and VGAT. LAMP5 labeling largely overlaps with VGAT immunoreactivity in both structures (arrowheads) (B) Quantitative evaluation of synaptic puncta shows that the vast majority of LAMP5 positive synapses co-express VGAT (n = 4 photomicrographs for GP and for OB). (C) Immunoisolation of vesicles using a VGAT antibody followed by western blotting. LAMP5 is specifically found in the VGAT positive fraction. Scale bar: 5 μm.

In *C*. *elegans*, the LAMP5 orthologue UNC-46 has been implicated in trafficking of the vesicular GABA-transporter UNC-47 to synaptic vesicles [14]. We investigated the relation of LAMP5 with the mouse orthologue of UNC-47, the vesicular GABA transporter VGAT. Immunofluorescence in both, the GP and the EPL of the OB, showed that expression of VGAT largely overlapped with LAMP5 (Fig 4A). Quantitative evaluation demonstrated that 76.8 +/- 2.4% and 87.1 +/-3.6% of all LAMP5 positive punctae co-labeled for VGAT in the OB and in the GP respectively (Fig 4B), indicating the presence of both molecules at the same synapses. We used synaptic vesicle-immunoisolation to investigate if both molecules were present in the same vesicles. Pull-down of the VGAT positive vesicles from the adult OB followed by western blotting demonstrated the presence of LAMP5 in the precipitated fraction (Fig 4C).

In conclusion, these data suggest that LAMP5 is expressed in subpopulations of GABAergic neurons where it largely co-localizes in synaptic vesicles with VGAT. Thus, expression and cellular localization of the protein was in agreement with a function in GABAergic synaptic transmission. However, expression of LAMP5 was restricted to specific inhibitory subpopulations in the striatum and the OB, but not pan-GABAergic, since GABAergic structures like SNr or GP were negative for the mRNA. This argued against a generalized role as an essential trafficking factor for VGAT.

**Fig 5:**
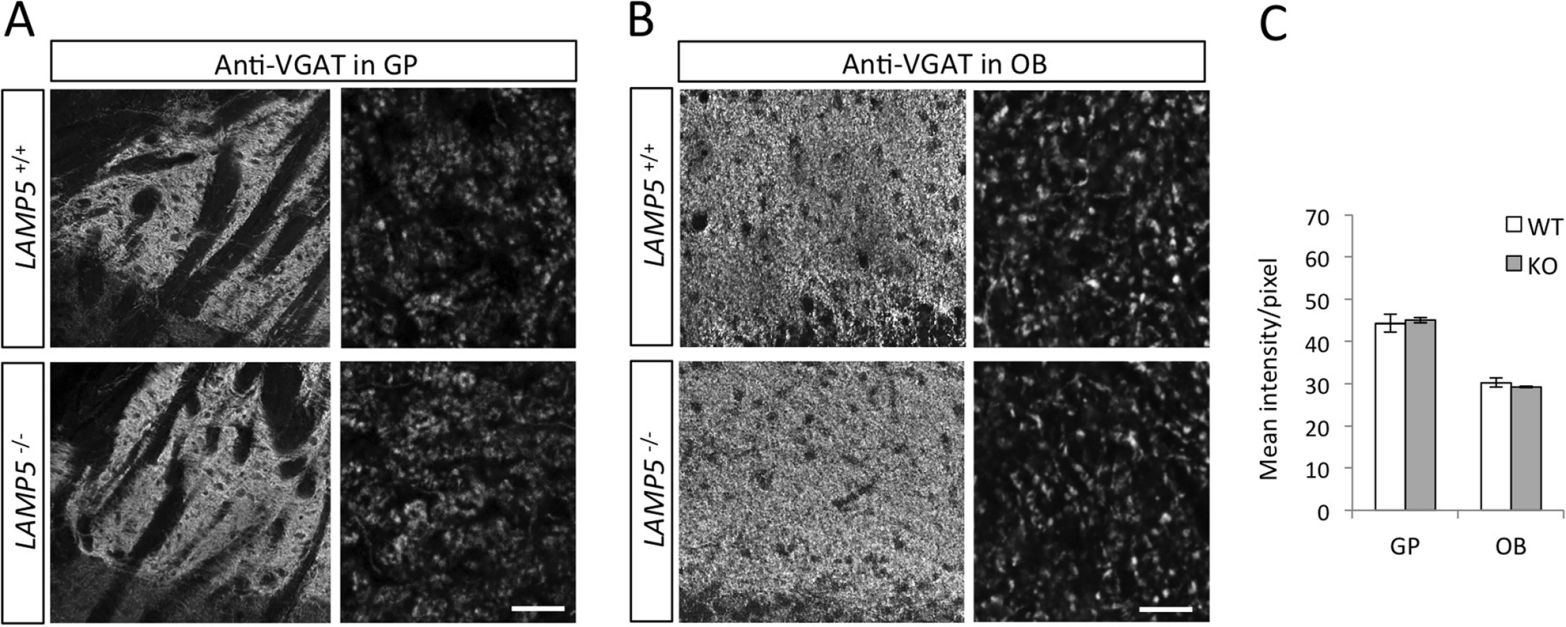
*LAMP5* knockout mice show normal VGAT distribution. (A, B) Immunostaining of LAMP5 and VGAT on GP (A) and OB (B) histological sections show no change in tissue and subcellular distribution in the absence of LAMP5. (C) Signal intensity of VGAT staining in the GP and in the OB, assessed by ImageJ analysis, is also unchanged. Mean intensity was calculated over 3 photomicrographs. Scale bar: A, 100 μm (left), 5 μm (right); C, 50 μm (left), 5 μm (right).

To further investigate this point we analyzed VGAT localization in the LAMP5-deficient brains. In both, the GP and the OB distribution and quantity of VGAT was unaffected by the absence of LAMP5 (Fig 5). Thus, LAMP5-deficiency in mice did not induce detectable changes in the sub-cellular localization of VGAT.

### Altered synaptic plasticity in the absence of LAMP5

**Fig 6:**
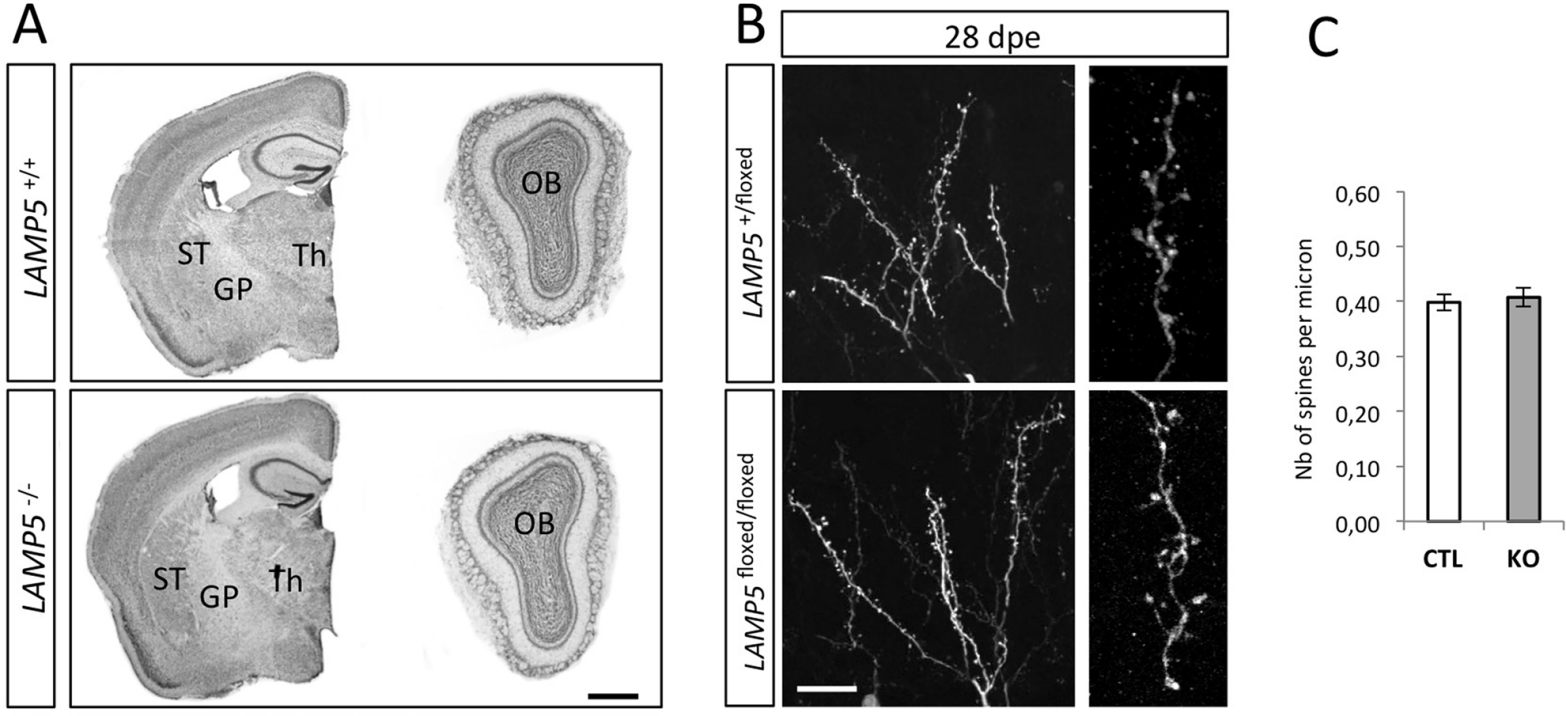
Absence of LAMP5 does not alter brain structure nor spine density in the OB. (A) Cresyl violet staining of forebrain and OB sections shows no obvious structural changes in the brain in the absence of LAMP5. (B) Dendrites of control (*LAMP5 flox/+*) and mutant (*LAMP5 flox/flox*) newly generated granule neurons observed in the EPL of the OB 28 days after their electroporation (28dpe) with GFP and Cre recombinase expression plasmids. (C) Quantification of spine density on the dendrites of control (CTL) and KO newly integrated neurons. Dendrite morphology and spine density are unchanged in *LAMP5* KO. Dendrites n = 26 (flox/+) and 27 (flox/flox). Statistics: Wilcoxon test p-value = 0.9645. Scale bars: A, 1mm (0.5 mm for the OB); B, 20 μm (left), 5 μm (right). ST: Striatum; Th: Thalamus.

Constitutively *LAMP5* knock out (KO) mice were born at Mendelian ratio and showed no gross morphological alterations. Histological observations of brain sections based on Nissl-staining revealed that overall brain structure was unaffected (Fig 6A). Moreover, the indistinguishable distribution of VGAT in GP and OB (Fig 5) indicated normal synaptic organization.

Therefore, we asked if LAMP5-deficiency induced a selective disadvantage leading to morphological alterations when KO neurons had to integrate in a competitive context, as demonstrated before in the case of the proteoglycan Agrin [28]. As mentioned above, during postnatal neurogenesis in the OB new interneurons are permanently generated by neural stem cells situated along the walls of the lateral ventricles. After their migration they integrate in the OB circuitry. Postnatal brain electroporation allows the efficient expression of transgenes in a subpopulation of these neuronal lineages (compare Fig 4C). We used this approach to create a situation in which individual *LAMP5* KO cells had to form synapses in a WT context. To do so we electroporated a CRE recombinase expressing plasmid (together with GFP reporter plasmid that is co-transfected in almost all cells, Boutin et al., 2008) at P0 in the ventricular wall of mice that were either heterozygous for the targeted *LAMP5* locus (+/floxed) or homozygous for the floxed allele (floxed/floxed). Twenty eight days later, offspring of the targeted cells were analyzed after their integration into the OB. These analyses demonstrated that neuronal morphology (Fig 6B) and spine density (Fig 6C) were indistinguishable among both genotypes. Thus, even in a competitive context LAMP5 deficiency did not lead to detectable morphological alterations.

**Fig 7:**
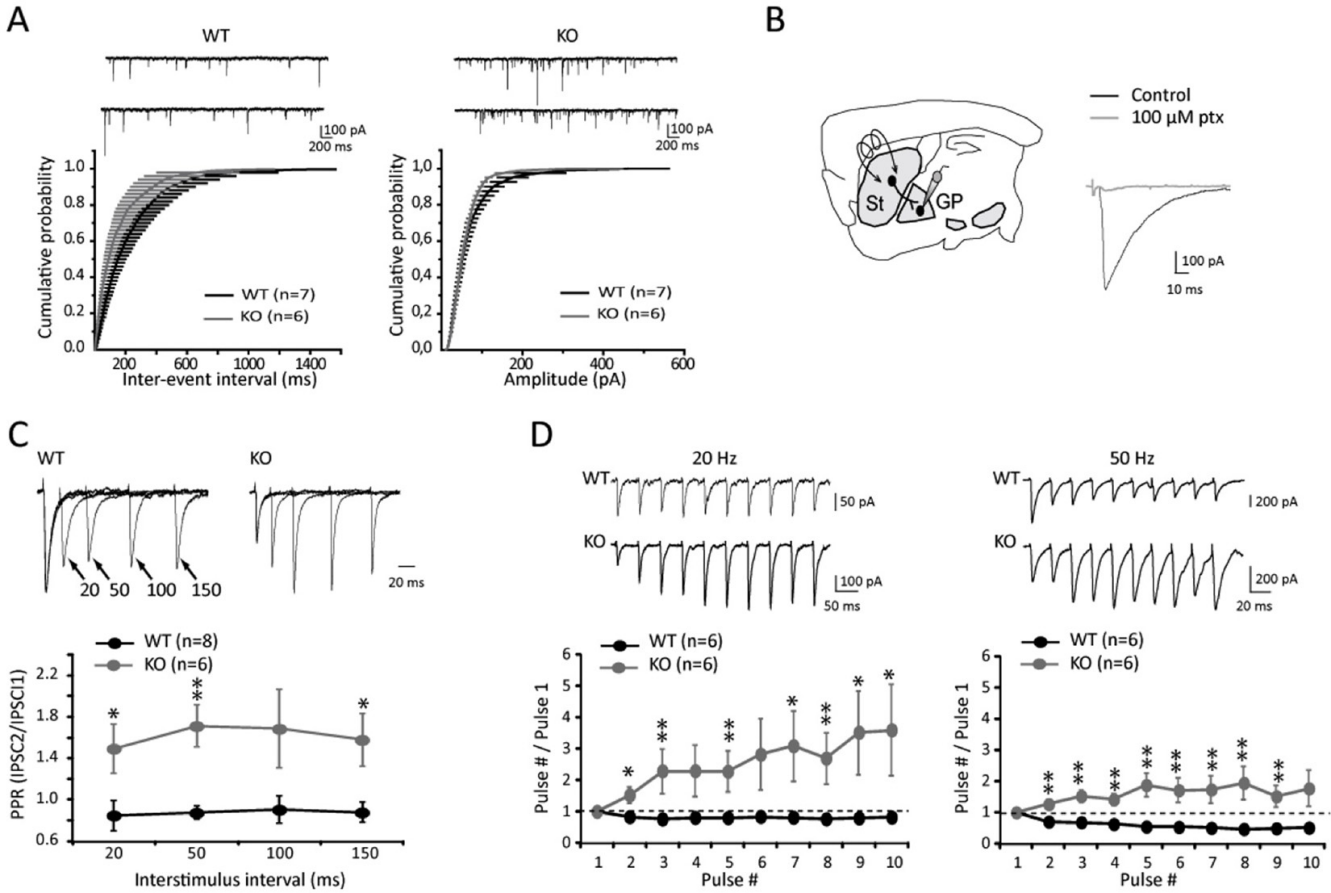
Short-term plasticity of the striatopallidal synapse is altered in *LAMP5* deficient mice. (A) Top: Representative traces of mIPSCs from WT and KO mice. Bottom: Cumulative inter-event interval (left) and amplitude (right) distributions of mIPSCs obtained in WT and KO mice (n = 400 events per cell). The frequency of mIPSCs is significantly increased in *LAMP5* KO compared to WT mice (*p* < 0.001; WT amplitude n = 3 mice, n = 4 slices, n = 7 cells / KO amplitude n = 2 mice, n = 4 slices, n = 5 cells; WT frequency n = 3 mice, n = 4 slices, n = 7 cells / KO frequency n = 2 mice, n = 4 slices, n = 6 cells). (B) Parasagittal section of the mouse brain showing the recording and stimulation sites. Evoked striatopallidal GABAergic IPSCs are blocked by the GABA_A_ receptor antagonist, picrotoxin (ptx). St: striatum, GP: globus pallidus. (C) Mean PPR values from WT and KO mice at different interstimulus intervals. Sample traces of PPR are shown above the graph (traces were scaled to first IPSCs). * p < 0.05, ** p < 0.01 vs. WT mice. (D) Synaptic depression during repeated stimulation (10 pulses at 20 and 50 Hz) in WT mice was replaced by facilitation in KO mice. Representative traces to 20 and 50 Hz trains are shown in WT and KO mice. * p < 0.05, ** p < 0.01 vs. WT mice. Error bars represent SEM.

Given the synaptic localization of LAMP5 and its proposed function as a VGAT transporter in *C*. *elegans*, we tested whether LAMP5 was important for synaptic physiology, thereby focusing on the striatopallidal synapse. We first quantified miniature inhibitory postsynaptic currents (mIPSCs) in GP neurons in the presence of 1 μM TTX to block activity-dependent release of neurotransmitter in WT and KO mice. While mIPSCs amplitudes were similar in WT and KO mice, we observed a statistically significant increase in the frequency of mIPSCs in KO mice (Fig 7A), suggesting a change in the presynaptic release apparatus. We then analyzed evoked IPSCs triggered by striatal stimulation in GP neurons. Such stimulation elicited inward IPSCs that were blocked by the GABA_A_ antagonist picrotoxin (Fig 7B). As LAMP5 is specifically expressed in striatal terminals, presynaptic function of the GABAergic striatopallidal synapse was investigated by using short-term plasticity protocols. The results showed that paired-pulse ratios (PPRs) of IPSCs, a standard measure of neurotransmitter release probability, were strongly affected in *LAMP5* KO mice. The moderate paired-pulse depression of striatopallidal synapses observed in WT mice was replaced by a strong facilitation in *LAMP5* KO mice at all four interstimulus intervals (Fig 7C). This suggests that deletion of LAMP5 decreases release probability, the most common cause for paired-pulse facilitation [29].

We then investigated the behavior of striatopallidal synapses during trains of stimuli at moderate frequency (10 pulses at 20 and 50 Hz). In these experiments synapses showed frequency-dependent short-term depression in WT mice (Fig 7D). Conversely, *LAMP5*-deficient mice exhibited a pronounced augmentation for the entire duration of the train, both at 20 and 50 Hz (Fig 7D). Altogether, these results suggest that LAMP5 appears critical for the regulation of spontaneous vesicular release and the dynamics of evoked GABAergic transmission, especially during periods of moderate activity.

### Behavioral analyses

**Fig 8:**
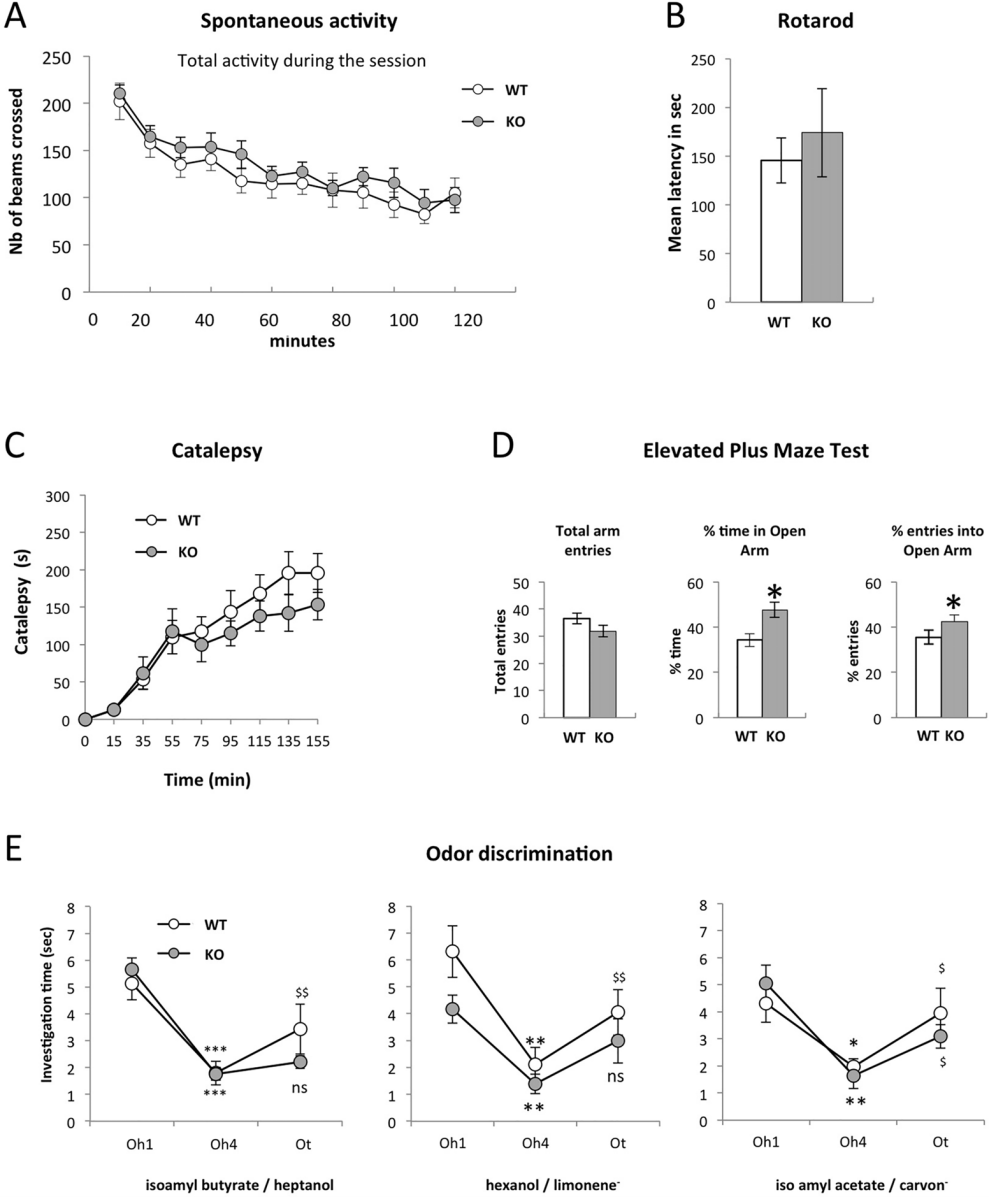
Absence of LAMP5 disrupts olfaction and leads to anxiety-like behavior. (A) Spontaneous activity of naive wild type (open circles) and KO mice (dark circles). Horizontal locomotor activity is recorded every 10 min for 2 hours. The exploration is similar over time between WT (n = 11) and KO (n = 16). (B) Motor coordination is assessed in a rotarod essay where mice are placed on a cylinder that rolls at increasing speed with time. The latency before the mice fall is measured. No difference is found between WT (n = 8) and KO (n = 7) mice. Kruskal-Wallis test p-value = 0.4875. (C) Catalepsy test. WT and KO mice are injected with haloperidol (i.p. 1mg/kg). The length of time the animals stay in a constrained position is recorded to a maximum of 300 seconds every 20 min. (WT n = 7; KO n = 7). ANOVA testing shows no genotype effect (F_1,13_ = 0.213, p-value = 0.652) and no interaction between time and genotype (F_7,91_ = 0.558; p = 0.788). (D) Elevated Plus Maze test. Left: Total number of entries in closed and open arms, an index of activity. Middle: percentage of time spent into the open arms. Right: percentage of open arm entries. The mutant mice are less anxious as they spend significantly more time exploring the open arms (Kruskal-Wallis test p-value = 0.01221) and enter preferentially into the open arms (Kruskal-Wallis test p-value = 0.03415; WT n = 11, KO n = 16). (E) Odor discrimination test. A first odorant (Oh: habituation odor) is presented for four consecutive trials followed by the presentation of a test odor (Ot). The time spent by the mice investigating the respective odorant is recorded. Habituation occurs similarly for both WT and KO mice as shown by a significant decrease in investigation time between the first (Oh1) and last (Oh4) trials (*, p<0.05; **, p<0.01; ***, p<0.001). Discrimination is partially impaired in KO mice as they do not discriminate between isoamyl butyrate and heptanol or between hexanol and limonene^-^ (Ot vs Oh4: $, p<0.05; $ $, p<0.01; ns: not significant). Statistics: Wilcoxon test. Number of animals: isoamyl butyrate/ heptanol WT n = 14, KO n = 27; hexanol/limonene WT n = 10, KO n = 13; isoamyl actetate/carvon WT n = 7, KO n = 7. Error bars represent SEM.

Finally, we used set of behavioral tests to investigate if loss of LAMP5 and the consequent deficits in synaptic transmission led to alterations at this level. Since the striatal projection systems, that expresses high levels of LAMP5 (Fig 1), is implicated in motor control, we first focused on this aspect. General locomotor activity and exploratory behavior were measured in an activity test, where animals were allowed to explore the test environment for two hours. As expected, total exploratory activity decreased over the test interval. Significant differences between control and *LAMP5* KO were not detectable (Fig 8A). We investigated motor coordination and endurance using a Rotarod test. Again, latency to fall off the rotating cylinder was not different between WT and *LAMP5* KO (Fig 8B). Thus, significant alterations in activity and motor coordination were not detectable in the absence of LAMP5.

LAMP5 is expressed in the axon terminals of two populations of striatal neurons that connect directly and indirectly (via two relays: GP and subthalamic nucleus, STN) the basal ganglia output structures SNr and EP. Striatal “direct” and “indirect” neurons exert opposite influence onto basal ganglia outflow, and consequently on motor function. Therefore, by affecting GABA transmission at synapses established by direct and indirect striatal neurons, LAMP5 deficiency *per se* might not disrupt the excitation/inhibition balance in the basal ganglia output structures. This is in agreement with the lack of motor deficits in KO mice under basal conditions. We tested the consequences of LAMP5 deficiency in a model of imbalanced activity between the two pathways. Striatal neurons of the direct pathway express predominantly D1 dopamine receptors while striatal neurons in the indirect pathway express preferentially D2 dopamine receptors (D2R). The antipsychotic haloperidol depresses motor function and induces a cataleptic state primarily attributed to the drug’s high potential to block striatal D2R (Sanberg Nature 1980, Wadenberg et al., 2001). According to the schemes of basal ganglia functioning (Albin et al., 1989), such blockade would increase striatal indirect neuron activity, unbalancing the system towards excitation of basal ganglia output structures and hence would result in inhibiting motor function. In this context, LAMP5-deficiency is expected to reduce haloperidol-induced catalepsy. We performed intra-peritoneal injection of haloperidol in KO (n = 12) and control (n = 8) mice but did not detect a statistically significant reduction in cataleptic state in mutants (Fig 8C).

Next, we performed the elevated Plus-maze test, a well-established assay of anxiety-related behavior in rodents [30] [31]. Here, WT and KO behaved not significantly different concerning total arm entries, in agreement with unaltered locomotor activity. However, mutants entered open arms more frequently and spent more time in these compartments (Fig 8D), suggesting decreased anxiety levels.

Finally, we tested olfactory capacities of *LAMP5*-deficient mice. Correct function of GABAergic inhibition by granule neurons in the OB has been shown to be crucial for efficient odor discrimination [32]. Given that OB granule neurons represent one of the few cell populations that express high LAMP5 levels at their synapses, we next tested odor discrimination using three pairs of odors (odor pair 1: isoamyl butyrate/ heptanol; odor pair 2: hexanol/limonene; odor pair 3: isoamyl actetate/carvon). All animals presented solid habituation to the first odor over four successive trials as demonstrated by ANOVA analyses showing a significant effect of trial number across habituation trials (odor pair 1: F_3,3_ = 51.807, p<0.001; odor pair 2: F_3,3_ = 41.536, p<0.001; odor pair 3: F_3,3_ = 16.99, p<0.001). Interaction between trials and genotype was not detected except for odor 2, due to a significant difference in investigation time during the first odor presentation (odor pair 1: F_3,117_ = 0.323, p = 0.809; odor pair 2: F_3,63_ = 5.142, p = 0.003; odor pair 3: F_3,48_ = 1.04, p = 0.383). Nonetheless, habituation occurred and the significant decrease in odor investigation time between the first (Oh1) and last (Oh4) trials was confirmed by Wilcoxon *post hoc* comparisons (Fig 8E). However, while wild type animals were in all situations able to distinguish the new test odor (Ot), *LAMP5* KO showed no significant discrimination for two out of three odors, heptanol and limonene- (Fig 8E).

In conclusion, *LAMP5*-deficient mice showed normal activity and motor behavior but had decreased anxiety levels. Moreover, in agreement with the restricted expression of LAMP5 protein at OB inhibitory synapses, we identified minor deficits in odor discrimination.

## Discussion

We found that LAMP5 is confined to sub-populations of GABAergic neurons where it shows synaptic localization. LAMP5-deficency in mice does not affect overall brain morphology or lead to detectable structural or connectivity changes in the analyzed LAMP5-positive neuron populations, but induces subtle alterations in synaptic plasticity and behavior.

In *C*. *elegans* the LAMP5 orthologue UNC-46 has been suggested to play an essential role in loading of GABA containing synaptic vesicles through the correct trafficking of VGAT [14]. This conclusion was based on several lines of evidence. First, *unc-46* is expressed by all 26 GABAergic neurons, which, however, develop normally in its absence. Second, the synaptically localized proteins VGAT and UNC-46, were mislocalized to non-synaptic compartments in the absence of the respective other protein, suggesting an interaction between both molecules that controls their sub-cellular targeting. Third, in *unc-46* mutants the frequency of GABA miniature currents was reduced by 90%, while amplitude of the remaining minis was normal. This indicates that in the absence of the protein a smaller number of synaptic vesicles is filled with neurotransmitter, but that these vesicles contain normal GABA concentrations. Lastly, overexpression of VGAT was able to rescue the locomotion defects induced by *unc-46* deficiency.

In agreement with the data in nematodes, our data supports a function of LAMP5 in GABAergic neurons in mice. The protein is confined to GABAergic synapses and GABAergic synaptic transmission is affected in its absence. However, it is also evident that this function is distinct from its supposed role in the worm. In both species, LAMP5 co-localizes with VGAT in GABAergic synaptic vesicles. But, unlike VGAT and other than in the worm, LAMP5 is not expressed by all GABAergic neurons, but is confined to subsets, arguing more for a specific regulatory role than for a general function in transporter trafficking. Moreover, while in the worm UNC-47 is miss-targeted in *unc-46* mutants, in the absence of LAMP5 in mice VGAT shows its normal localization in brain structures that normally co-express both molecules, like the GP and the OB. This, again, argues against a general role of LAMP5 in transporter trafficking from ER to the synapse and might be indicative of a tailor-made function potentially affecting VGAT activity in a subset of synaptic vesicles or at a specific time of GABA release.

Furthermore, while activity-independent release of GABA is severely affected in *unc-46* mutants, our data in KO mice implicate LAMP5 in a more subtle regulation of inhibitory synaptic transmission. When recording GP neurons, the main target of striatal terminals expressing high level of LAMP5, we found that the frequency of mIPSCs was not decreased but increased in LAMP5 knockout mice while amplitude distribution was not changed. Surprisingly, the transition from moderate paired-pulse depression in WT mice to facilitation in *LAMP5* knock-out mice indicates that the deletion of LAMP5 decreases the release probability at striatopallidal synapses. Short-term plasticity of these synapses was also affected in the absence of LAMP5, switching from depression in WT to augmentation in *LAMP5* knockout mice, when responding to train stimulation. Altogether, these observations suggest that LAMP5 acts in the regulation of GABAergic transmission at the presynaptic level and that activity-independent and evoked release processes are differentially dependent on its presence. Numerous mechanisms contribute to short-term plasticity including regulation of vesicle availability and calcium homeostasis in nerve terminals (Fioravante and Regehr, 2011). Although it is difficult to predict at this point which steps of the release machinery are modulated by LAMP5, our results highlight a critical role for this protein in regulating GABAergic transmission in the central nervous system. Consistent with such a critical function in fine-tuning specific GABAergic synapses, *LAMP5* KO mice show subtle behavioral abnormalities, as evidenced by decreased anxiety and altered odor discrimination. This correlates with LAMP5 expression in the ventral pallidum, that processes motivational and emotional information, and in OB granule cells that are implicated in odor discrimination (Shepherd, Greer, 2007).

While our data points clearly to a regulatory function of LAMP5 in induced synaptic transmission in subsets of GABAergic synapses, more detailed cell biological analyses are hampered by the lack of suitable cellular models. High-resolution analyses depend on the availability of in vitro models that can be studied in terms of protein interactions and localization. LAMP5 is expressed only at late stages of brain development, when synapses mature and start to function [13]. We observed that LAMP5 expression is never induced in cultured olfactory bulb primary neurons, suggesting that even when synaptic contacts are formed induction of LAMP5 expression might depend either on the presynaptic activity of the GABA neuron or on specific retrograde signals from the other components of the synapse: the post-synaptic neuron or the glial cells surrounding the functional synapse.

Given the paucity of suitable neuronal in vitro models, most information about the cellular function of LAMP5 relies on observations in the human immune system and on gain-of-function studies in non-neuronal HeLa cells. In the human immune system LAMP5 is expressed at high levels in plasmacytoid dendritic cells (pDCs), where it allows discrimination of normal from leukemic pDCs in tissue and blood [17]. At the molecular level, human pDCs express high amounts of UNC-93B1, a protein implicated in toll-like receptor (TLR) trafficking, which is related to the *C*. *elegans* protein F31D5.2. The latter factor has been identified as a direct binding partner for LAMP5 using two-hybrid studies in the worm [33].

Interestingly, gain-of-function studies in HeLa cells demonstrated that LAMP5 is, when expressed alone, predominantly present at the cell membrane while UNC-93B1 accumulates in the ER. Co-expression of both molecules induces their redistribution into LAMP1-positive endosomes, altogether indicating that they function as co-chaperones that reciprocally influence their intracellular addressing (Defays et al., 2011). Therefore, while our data results argue against a major role in trafficking of the GABA-transporter VGAT to synaptic vesicles, a role of LAMP5 as a trafficking chaperone for other cargo or to reach other organelles could still be contemplated.

Another important observation concerns the regulation of LAMP5 protein expression. Highest mRNA levels are observed in the neo- and piriform cortex, the hippocampal CA1/CA3 regions and the mitral cells of the OB. All these populations contain predominantly glutamatergic neurons. However, none of these populations expresses considerable amounts of LAMP5 protein, neither in their cell bodies nor in their synapses. How could this strict suppression of protein production be achieved? One typical mechanism for posttranslational regulation represents microRNA targeting the mRNA. However, scanning the LAMP5 mRNA for potential microRNA binding sites revealed only one potential target sequence for microRNAs that so far has not been involved in brain development and function. Moreover, in general microRNAs modulate expression levels of a given target gene, but do not induce a total suppression, as we see in the case on LAMP5 in glutamatergic neurons. Therefore, we do not favor such a mechanism. A more likely explanation might lie in the potential function of LAMP5 as a trafficking chaperone in GABAergic synapses. In the absence of suitable cargo, LAMP5 might well be targeted to the lysosome and degraded.

In conclusion, we describe the expression of Lamp5 at high resolution and its molecular context. Functional analysis based on conditionally Lamp5 knockout mice indicate a subtle but detectable role in GABAergic synapse function, leading to consequences at the behavioral level.

## References

[1] Schmieg N, Menendez G, Schiavo G, Terenzio M (2014) Signalling endosomes in axonal transport: travel updates on the molecular highway. Semin Cell Dev Biol 27: 32–43. 10.1016/j.semcdb.2013.10.004 24171925

[2] Sudhof TC, Rizo J (2011) Synaptic vesicle exocytosis. Cold Spring Harb Perspect Biol 3. 10.1101/cshperspect.a005637 PMC3225952 22026965

[3] Castillo PE, Chiu CQ, Carroll RC (2011) Long-term plasticity at inhibitory synapses. Curr Opin Neurobiol 21: 328–338. 10.1016/j.conb.2011.01.006 21334194 PMC3092861

[4] Saheki A, Seki J, Nakanishi T, Tamai I (2012) Effect of back pressure on emulsification of lipid nanodispersions in a high-pressure homogenizer. Int J Pharm 422: 489–494. 10.1016/j.ijpharm.2011.10.060 22108638

[5] Saheki T, Inoue K, Ono H, Katsura N, Yokogawa M, et al (2012) Effects of supplementation on food intake, body weight and hepatic metabolites in the citrin/mitochondrial glycerol-3-phosphate dehydrogenase double-knockout mouse model of human citrin deficiency. Mol Genet Metab 107: 322–329. 10.1016/j.ymgme.2012.07.021 22921887

[6] Saheki Y, De Camilli P (2012) Synaptic vesicle endocytosis. Cold Spring Harb Perspect Biol 4: a005645 10.1101/cshperspect.a005645 22763746 PMC3428771

[7] Maycox PR, Link E, Reetz A, Morris SA, Jahn R (1992) Clathrin-coated vesicles in nervous tissue are involved primarily in synaptic vesicle recycling. J Cell Biol 118: 1379–1388. 1325974 10.1083/jcb.118.6.1379 PMC2289614

[8] Blondeau F, Ritter B, Allaire PD, Wasiak S, Girard M, et al (2004) Tandem MS analysis of brain clathrin-coated vesicles reveals their critical involvement in synaptic vesicle recycling. Proc Natl Acad Sci U S A 101: 3833–3838. 15007177 10.1073/pnas.0308186101 PMC374330

[9] Poudel KR, Bai J (2014) Synaptic vesicle morphology: a case of protein sorting? Curr Opin Cell Biol 26: 28–33. 10.1016/j.ceb.2013.09.001 24529243 PMC4079907

[10] Eskelinen EL, Schmidt CK, Neu S, Willenborg M, Fuertes G, et al (2004) Disturbed cholesterol traffic but normal proteolytic function in LAMP-1/LAMP-2 double-deficient fibroblasts. Mol Biol Cell 15: 3132–3145. 15121881 10.1091/mbc.E04-02-0103 PMC452571

[11] Eskelinen EL, Tanaka Y, Saftig P (2003) At the acidic edge: emerging functions for lysosomal membrane proteins. Trends Cell Biol 13: 137–145. 12628346 10.1016/s0962-8924(03)00005-9

[12] Saftig P, Tanaka Y, Lullmann-Rauch R, von Figura K (2001) Disease model: LAMP-2 enlightens Danon disease. Trends Mol Med 7: 37–39. 11427988 10.1016/s1471-4914(00)01868-2

[13] David A, Tiveron MC, Defays A, Beclin C, Camosseto V, et al (2007) BAD-LAMP defines a subset of early endocytic organelles in subpopulations of cortical projection neurons. J Cell Sci 120: 353–365. 17215451 10.1242/jcs.03316

[14] Schuske K, Palfreyman MT, Watanabe S, Jorgensen EM (2007) UNC-46 is required for trafficking of the vesicular GABA transporter. Nat Neurosci 10: 846–853. 17558401 10.1038/nn1920

[15] Tiveron MC, Hirsch MR, Brunet JF (1996) The expression pattern of the transcription factor Phox2 delineates synaptic pathways of the autonomic nervous system. J Neurosci 16: 7649–7660. 8922421 10.1523/JNEUROSCI.16-23-07649.1996 PMC6579082

[16] Boutin C, Hardt O, de Chevigny A, Core N, Goebbels S, et al (2010) NeuroD1 induces terminal neuronal differentiation in olfactory neurogenesis. Proc Natl Acad Sci U S A 107: 1201–1206. 10.1073/pnas.0909015107 20080708 PMC2824315

[17] Defays A, David A, de Gassart A, De Angelis Rigotti F, Wenger T, et al (2011) BAD-LAMP is a novel biomarker of nonactivated human plasmacytoid dendritic cells. Blood 118: 609–617. 10.1182/blood-2010-11-319699 21642595

[18] Dumoulin A, Rostaing P, Bedet C, Levi S, Isambert MF, et al (1999) Presence of the vesicular inhibitory amino acid transporter in GABAergic and glycinergic synaptic terminal boutons. J Cell Sci 112 (Pt 6): 811–823. 10036231 10.1242/jcs.112.6.811

[19] Beurrier C, Bonvento G, Kerkerian-Le Goff L, Gubellini P (2009) Role of glutamate transporters in corticostriatal synaptic transmission. Neuroscience 158: 1608–1615. 10.1016/j.neuroscience.2008.11.018 19063944

[20] Beurrier C, Lopez S, Revy D, Selvam C, Goudet C, et al (2009) Electrophysiological and behavioral evidence that modulation of metabotropic glutamate receptor 4 with a new agonist reverses experimental parkinsonism. FASEB J 23: 3619–3628. 10.1096/fj.09-131789 19525404

[21] Revest JM, Dupret D, Koehl M, Funk-Reiter C, Grosjean N, et al (2009) Adult hippocampal neurogenesis is involved in anxiety-related behaviors. Mol Psychiatry 14: 959–967. 10.1038/mp.2009.15 19255582

[22] Boulay D, Depoortere R, Oblin A, Sanger DJ, Schoemaker H, et al (2000) Haloperidol-induced catalepsy is absent in dopamine D(2), but maintained in dopamine D(3) receptor knock-out mice. Eur J Pharmacol 391: 63–73. 10720636 10.1016/s0014-2999(99)00916-4

[23] Boulay D, Depoortere R, Perrault G, Sanger DJ (2000) Decreased locomotor activity after microinjection of dopamine D2/D3 receptor agonists and antagonists into lobule 9/10 of the cerebellum: a D3 receptor mediated effect? Prog Neuropsychopharmacol Biol Psychiatry 24: 39–49. 10659982 10.1016/s0278-5846(99)00079-2

[24] Moreno MM, Linster C, Escanilla O, Sacquet J, Didier A, et al (2009) Olfactory perceptual learning requires adult neurogenesis. Proc Natl Acad Sci U S A 106: 17980–17985. 10.1073/pnas.0907063106 19815505 PMC2764902

[25] Schwenk F, Baron U, Rajewsky K (1995) A cre-transgenic mouse strain for the ubiquitous deletion of loxP-flanked gene segments including deletion in germ cells. Nucleic Acids Res 23: 5080–5081. 8559668 10.1093/nar/23.24.5080 PMC307516

[26] Whitman MC, Greer CA (2009) Adult neurogenesis and the olfactory system. Prog Neurobiol 89: 162–175. 10.1016/j.pneurobio.2009.07.003 19615423 PMC2748178

[27] Boutin C, Diestel S, Desoeuvre A, Tiveron MC, Cremer H (2008) Efficient in vivo electroporation of the postnatal rodent forebrain. PLoS One 3: e1883 10.1371/journal.pone.0001883 18382666 PMC2270900

[28] Burk K, Desoeuvre A, Boutin C, Smith MA, Kroger S, et al (2012) Agrin-signaling is necessary for the integration of newly generated neurons in the adult olfactory bulb. J Neurosci 32: 3759–3764. 10.1523/JNEUROSCI.4906-11.2012 22423096 PMC3313839

[29] Zucker RS, Regehr WG (2002) Short-term synaptic plasticity. Annu Rev Physiol 64: 355–405. 11826273 10.1146/annurev.physiol.64.092501.114547

[30] Pellow S, Chopin P, File SE, Briley M (1985) Validation of open:closed arm entries in an elevated plus-maze as a measure of anxiety in the rat. J Neurosci Methods 14: 149–167. 2864480 10.1016/0165-0270(85)90031-7

[31] Lister RG (1987) The use of a plus-maze to measure anxiety in the mouse. Psychopharmacology (Berl) 92: 180–185. 3110839 10.1007/BF00177912

[32] Gheusi G, Cremer H, McLean H, Chazal G, Vincent JD, et al (2000) Importance of newly generated neurons in the adult olfactory bulb for odor discrimination. Proc Natl Acad Sci U S A 97: 1823–1828. 10677540 10.1073/pnas.97.4.1823 PMC26520

[33] Li S, Armstrong CM, Bertin N, Ge H, Milstein S, et al (2004) A map of the interactome network of the metazoan C. elegans. Science 303: 540–543. 14704431 10.1126/science.1091403 PMC1698949

